# Thanksgiving

**Published:** 1858-03

**Authors:** 


### THANKSGIVING.

AN educated and efficient clergyman writes: "I should have ordered your Journal before this; but the inclosed dollar is the first one I could conveniently spare. I know well, and by painful experience, the evils of poverty and sickness. I can fully appreciate "Clerical Letters" of the September number.

"As soon as I can spare five dollars, I will send for the four back volumes. But, as I have a large family (and I thank the Lord for it) and a small salary, with large poverty, I cannot tell when I shall be able to spare the money."

It might seem rather a doubtful cause of thankfulness on the part of a poor man. But this gentleman was right; each member of a loving family is a separate spring of pure delight to a parent, and helps to lighten troubles which otherwise would, and do, daily crush many hearts. To be growing old, and have no children or grandchildren, presents a bleaker prospect than to be perched on a pyramid of the desert, or upon a glacier of the Frozen Sea. Single folks, marry—and marry while you are young!

